# Adverse Pathological Findings at Radical Prostatectomy following Active Surveillance: Results from the Movember GAP3 Cohort

**DOI:** 10.3390/cancers14153558

**Published:** 2022-07-22

**Authors:** Cristina Marenghi, Zhuyu Qiu, Jozien Helleman, Daan Nieboer, Josè Rubio-Briones, Peter R. Carroll, Lui Shiong Lee, Riccardo Valdagni, Paul C. Boutros, Nicola Nicolai, on behalf of Movember Foundation’s Global Action Plan Prostate Cancer Active Surveillance (GAP3) Consortium

**Affiliations:** 1 Prostate Cancer Program, Fondazione IRCCS Istituto Nazionale dei Tumori, 20133 Milan, Italy; riccardo.valdagni@unimi.it or; 2 Department of Human Genetics, University of California Los Angeles, Los Angeles, CA 90095, USA; zqiu@mednet.ucla.edu; 3 Department of Urology, University of California Los Angeles, Los Angeles, CA 90095, USA; 4 Jonsson Comprehensive Cancer Centre, University of California Los Angeles, Los Angeles, CA 90095, USA; 5 Institute for Precision Health, University of California Los Angeles, Los Angeles, CA 90095, USA; 6 Department of Urology, Erasmus MC Cancer Institute, University Medical Center, 3015 GD Rotterdam, The Netherlands; j.helleman@erasmusmc.nl (J.H.); d.nieboer@erasmusmc.nl (D.N.); 7 Department of Public Health, Erasmus MC Cancer Institute, University Medical Center, 3015 GD Rotterdam, The Netherlands; 8 Department of Urology, Hospital VITHAS 9 de Octubre, 46025 Valencia, Spain; jrubio@clinicadoctorrubio.es; 9 Department of Urology, University of California, San Francisco, CA 94158, USA; peter.carroll@ucsf.edu; 10 Department of Urology, Sengkang General Hospital and Singapore General Hospital, Singapore 544886, Singapore; Lee.Lui.shiong@singhealth.com.sg; 11 Department of Oncology and Hemato-Oncology, Università Degli Studi di Milano, 20132 Milan, Italy; 12 Department of Radiation Oncology, Fondazione IRCCS Istituto Nazionale dei Tumori, 20133 Milan, Italy; 13 Urology Unit, Fondazione IRCCS Istituto Nazionale dei Tumori, 20133 Milan, Italy; nicola.nicolai@istitutotumori.mi.it

**Keywords:** active surveillance, outcome, pathology, prostatectomy, classification, prostatic neoplasms, prognosis, risk assessment, watchful waiting

## Abstract

**Simple Summary:**

Active surveillance (AS) is a standard option for low-risk prostate cancer patients wishing to preserve their quality of life by avoiding or delaying radical treatment side effects. We investigated the consequences of postponing radical prostatectomy (RP) according to stringent or more expansive criteria at inclusion in active surveillance. Features at radical prostatectomy of men withdrawn from AS showed that most of them still have favorable pathology. Frequency of unfavorable pathology was associated with wider entry criteria, PSA density (PSAD) and age, and time spent in active surveillance. Nonetheless, they are restricted to local tumor extension and positive surgical margins, but do not include tumor grade or lymph node involvement. The prognostic implications of these findings remain uncertain, and a longer follow-up is needed.

**Abstract:**

Background: Little is known about the consequences of delaying radical prostatectomy (RP) after Active Surveillance (AS) according to stringent or wider entry criteria. We investigated the association between inclusion criteria and rates, and timing of adverse pathological findings (APFs) among patients in GAP3 cohorts. Methods: APFs (GG ≥ 3, pT ≥ 3, pN > 0 and positive surgical margins [R1]) were accounted for in very low-risk (VLR: grade group [GG] 1, cT1, positive cores < 3, PSA < 10 ng/mL, PSA density [PSAD] < 0.15 ng/mL/cm^3^) and low-risk (LR: GG1, cT1-2, PSA ≤ 10 ng/mL) patients undergoing subsequent RP. The Kaplan–Meier method and log–rank test analyzed APF-free survival. Stratified mixed effects models analyzed association. Results: Out of 21,169 patients on AS, 1742 (VLR: 721; LR: 1021) underwent delayed RP. Most (60.8%) did not have APFs. APFs occurred more frequently (44.6% vs. 31.7%; OR 1.54, *p* < 0.001) and earlier (median time: 40.3 vs. 62.6 months; *p* < 0.001) in LR patients, and consisted of pT ≥ 3 (OR 1.47, *p* = 0.013) or R1 (OR 1.80, *p* < 0.001), but not of GG ≥ 3 or node involvement. Age (OR 1.05, *p* < 0.001), PSAD (OR 23.21, *p* = 0.003), and number of positive cores (OR 1.16, *p* = 0.004) were independently associated with APFs. Conclusions: AS stands as a safe option for low-risk patients, and most do not have APFs at surgery. Wider entry criteria are associated with pT3 and R1. The prognostic implications remain uncertain.

## 1. Introduction

Prostate cancer (PCa) is the second most incident cancer amongst men worldwide. More than 1,400,000 new cases per year occur globally. The highest rates are recorded in Northern and Western Europe, the Caribbean, Australia/New Zealand, Northern America, and Southern Africa [1]. Many men with early PCa show a long and indolent natural history of disease [2]. Therefore, treating all men would result in substantial overtreatment, exposing them to the side effects and costs of active management [3].

The purpose of active surveillance (AS) is to reduce overtreatment by avoiding active therapy of indolent cancer or postponing curative treatment up to the occurrence of disease progression. Most world-wide AS protocols include patients with low-risk PCa and assume disease progression as tumor size increasing or as a worsening in the biopsy score (upgrading of the Grade Group) [4]. Nonetheless, the outcomes and variables interaction in categories of low-risk patients have not been extensively studied. Three randomized clinical trials comparing watchful waiting or active monitoring and radical treatments (prostatectomy or external beam radiotherapy) in men with early PCa identified small or no significant differences in survival endpoints [5,6,7].

The main study aim is to investigate how the treatment delay could differently affect the outcome for those patients discontinuing AS. We focus on men with Grade Group (GG) 1 PCa enrolled in AS and compare different inclusion criteria (LR sub-group vs. VLR sub-group). Adverse pathological findings (APFs) at definitive pathology of subsequent radical prostatectomy (RP) were chosen as prognostic endpoints.

## 2. Materials and Methods

In 2014, the Movember Consortium started the GAP3 initiative to promote and accelerate research on AS in PCa patients (https://gap3.movemberprojects.com, accessed on 12 July 2022). A centralized database was created to share data from worldwide AS cohorts. GAP3 requirements included the recruitment of a minimum of 50 patients per year and an active patients’ registry for two years. Each participating center had an active registry of AS patients and ethical approval for sharing digital patient data in a centralized global database. Medical ethical approval and patient consent for AS were managed in compliance with local requirements by each registry. GAP3 database version “gap3data_3.2” was queried for this project.

At the time of the present analysis, 27 Institutions had provided data. Clinical and demographic characteristics at diagnosis, clinical follow-up, and reasons for AS discontinuation and subsequent treatments were collected. Cohorts’ monitoring strategy included regular prostate-specific antigen (PSA) testing, digital rectal examination, and repeat biopsies, which may have triggered radical treatment whenever disease re-classification or progression occurred. Multiparametric MRI was not routinely used prior to biopsy at the time of patient recruitment, and this prevents us from conducting an adequate analysis.

Patients who dropped out from AS for any reason undergoing radical prostatectomy (RP), alone or in combination with adjuvant treatments (radiation therapy, androgen deprivation therapy, or both), were identified. Those fulfilling all criteria for very low risk (VLR) and low risk (LR) sub-groups were considered for the analyses. VLR criteria included PSA < 10 ng/mL, clinical stage T1, Gleason Score (GS) 3 + 3, corresponding to ISUP 2016 GG 1 adenocarcinoma, up to two cores containing cancer, and PSA density (PSAD) < 0.15 ng/mL/cm^3^. Information about cancer involvement per core was mostly unavailable and was not included in this analysis. LR criteria included patients having PSA ≤ 10 ng/mL, clinical stage T1 or T2 (as for PRIAS criteria [8]), GG1 adenocarcinoma on biopsy, and no limitation in PSAD or number of positive cores were made. Those patients with VLR criteria have not been accounted for within the LR sub-group. Patients lacking any criterion were excluded from the analyses.

AS discontinuations were categorized as in the GAP3 database: “protocol reasons”, “without evidence of progression” and “other/unknown reasons”. “Protocol reasons” included clinical progression, pathological progression (GG > 1 and/or increase in number of cancer-containing biopsy cores, according with drop-out criteria adopted by each site), PSA progression (PSA-doubling time < 3 years or other PSA kinetics, as by individual center), and radiologic progression. Drop-outs “without evidence of progression” included patient or physician choices. “Other reasons” included any other or unknown reasons.

AS duration and time to eventual APF were calculated as the interval elapsed between the date of diagnosis and the date of surgery.

A central review of biopsy pathology was not performed. About 5% of patients only had data on multi-parametric MRI and no inference was performed according to this information.

Descriptive statistics were used for patients’ population characteristics at diagnosis.

APFs rates from definitive pathology were compared between the sub-groups. Patients were deemed to have an APF if they experienced any of the following: GS ≥ 4 + 3 (GG ≥ 3), pT ≥ 3, positive surgical margins, and lymph nodes involvement.

Differences between LR and VLR were assessed by the Student’s t-test and Fisher’s Exact test. The Kaplan–Meier method and log–rank test were used to analyze APF-free survival between the sub-groups. Univariate and multivariate mixed effects models estimated odds ratios (ORs) and 95% confidence intervals (CIs) for the association between APFs and preoperative patients risk factors, including: age, PSA, PSAD, clinical T-stage, number of positive cores, Charlson Comorbidity Index, ethnicity, smoking habit, and reasons for AS discontinuation. In all the models, individual site cohorts were used as stratified levels. Clinically significant variables are included in the final model. Odds ratios were also used to measure the association between biopsy schedules and APFs in stratified mixed effect models. All the tests were two-sided and *p* < 0.05 was considered significant.

Statistical analyses were performed using R version 3.6.0. Survival analysis was performed using ‘survival’ (version 3.1.12), ‘BoutrosLab.statistics.survival_0.4.20′ and ‘BoutrosLab.Plotting.survival_3.0.10′ packages in R (version 3.6.0, R Foundation for Statistical Computing, Vienna, Austria).

Each institution achieved IRB approval and agreement for sharing digital patient data.

## 3. Results

At the time of the analysis, the GAP3 database included 21,169 men enrolled in AS protocols. The entirety of this database comprised patients with low or favorable intermediate risk PCa who underwent AS between 1990 and 2018 (rate of recruitment per year is shown in supplementary Figure S2; median 2012, IQR 2009, 2015). In the whole cohort, the median time in surveillance was 28.8 months (range 0–257.3, IQR 14.0, 59.2).

**Figure 1:**
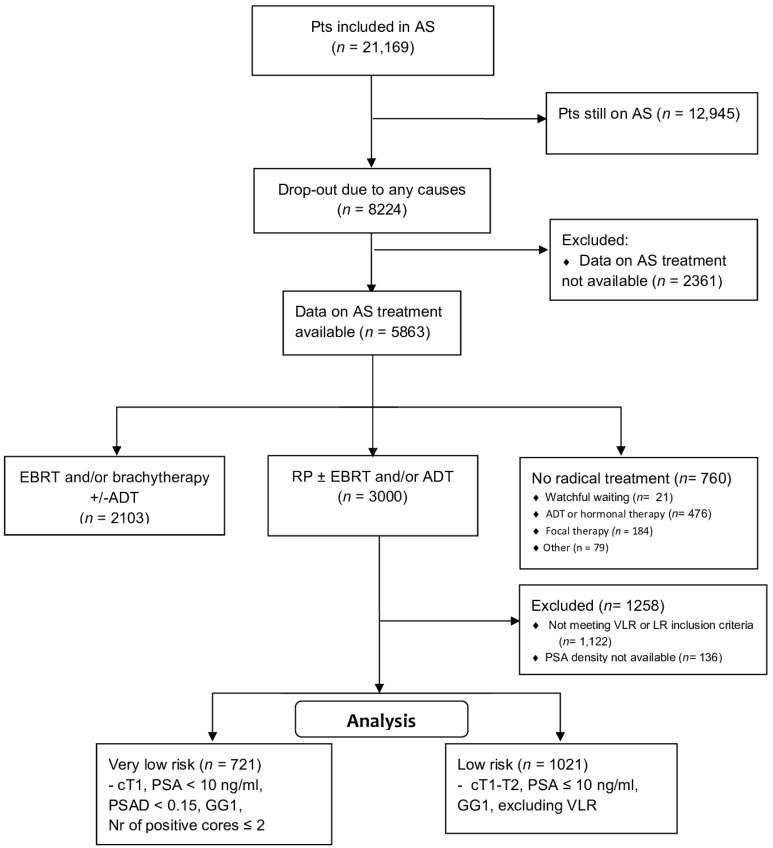
CONSORT flow diagram showing the selection process of the study population from the whole GAP3 AS cohort.

A CONSORT diagram reports on the selection process for this analysis (Figure 1). Overall, 5863/21,169 patients (27.7%) dropped out and were actively managed: 3000 (51.2%) underwent RP. The median time between diagnosis and surgery was 19.3 months (95% CI, 12.6, 37.4, range: 0.1–222 months). Supplementary Tables S1 and S2 report the baseline and definitive pathology of all patients undergoing surgery. The target population included 1742 patients with complete data: 721 (41.4%) were VLR and 1021 (58.6%) were LR patients, respectively.

Table 1 depicts the characteristics of the patients stratified by risk sub-group at diagnosis. LR patients were significantly younger and had significantly higher median PSA values. As expected, PSAD was significantly higher in LR cases. PSAD distribution is shown in supplementary Figure S3. As by entry criteria, cT2 cases (25.8%) and patients having more than two cancer-containing cores (12.6%) were all LR. Most patients had missing information for Charlson Comorbidity Index, ethnicity, and smoking habit. No significant differences were found according to reasons for AS discontinuation between the two sub-groups. Most patients (60.4%) abandoned AS due to protocol reasons, 7.2% dropped out without evidence of progression (mainly due to patient or physician decision), and 32.4% men stopped AS for unknown reasons (Table 1). Proportions were similar between these patients and those undergoing radiation therapy as a primary rescue treatment. The characteristics of the latter are described in Supplementary Table S3.

Out of the total of 1742 prostatectomies, APFs were reported in 683 (39.2%): 228 (31.7%) in the VLR and 455 (44.6%) in the LR sub-group, respectively. The median time to APF was 62.6 months (95% CI: 55.4–71.8) in the VLR sub-group and 40.3 months (95% CI: 36.3–44.1) in the LR sub-group (*p* < 0.001; log–rank test).

Table 2 depicts the different APFs features according to sub-groups. Patients in the LR group have about 50% higher probability of APF, compared with men in the VLR group. Of note, the differences are significant for pT category and positive surgical margins, but not for GG ≥ 3 (GS ≥ 4 + 3) or lymph node involvement. Differences are not found according to each GG (from 3 to 5).

Univariable and multivariable analyses (Table 3A,B) assessed that LR patients show a significantly higher risk of APFs. Elderly patients have higher probabilities of APFs (*p* < 0.001). The median time to event is 73.9 months for those younger than 55 years and 29.1 months for those between 71 and 80 years.

Mixed effect models have been used to investigate variables which might be associated with APF (Table 3C). In multivariable models, elder age, higher PSAD, and a higher number of containing-cancer biopsy cores at diagnosis have been shown to associate with APFs. Patients undergoing RP without clinical progression (*p* = 0.061) or unknown/other reasons display a lower risk of APF (*p* = 0.031).

### Adverse Pathology and Timing of Re-Biopsy at Follow-Up

Complete data regarding repeat biopsies during AS were available in 1324 (76%) of cases. Descriptive statistics of number and timing of biopsy during AS are reported in Supplementary Table S4. The majority had their first surveillance biopsy within 1 year since diagnosis (70.1%), and most of them (83%) had just 1 or 2 biopsy sets. Compared to LR, VLR patients underwent their first re-biopsy slightly later, had less frequently cancer detection in their first re-biopsy, and had fewer biopsies during follow-up.

Those patients having cancer in their first re-biopsy underwent more biopsy sets than those without cancer (median 1, IQR 1–2 vs. median 2, IQR 1–3; *p* < 0.001; Mann–Whitney U test). A positive trend between the number of biopsy sets and APFs was found in all sub-groups (Supplementary Figure S1).

## 4. Discussion

Although clinical evidence supports the safety and efficacy of AS in low and very-low PCa, as metastases are very rare and 15-years cancer-specific survivals range from 94–100% [9,10,11,12], some clinical issues still need to be unraveled. For instance, it remains unclear whether the precise entry criteria and time on AS influence the oncologic outcome for men who progress to treatment.

In the GAP3 data, most patients undergoing RP had an organ-confined disease with favorable features at definitive pathology (60.8%). However, a significantly higher rate of APFs was found among patients undergoing surgery after disease progression than patients undergoing surgery without or unknown disease progression. Nonetheless, the high proportion of favorable features at definitive pathology may lead to the consideration that AS exit criteria are still imperfect in preventing overtreatment. Patients in the LR sub-group underwent RP earlier than VLR patients and displayed a significantly higher rate of APFs, consisting of greater pathological T stage and rate of positive margins, but not of worst Grade Group; no difference was found in lymph node involvement as well, possibly due to small numbers. APFs tended to occur earlier among LR than VLR patients. The reasons for this time delay remain unclear and may depend on tumor biology or on different surveillance strategies (Supplementary Table S4).

Previous experiences addressing outcomes according to risk stratification in GG1 patients undergoing delayed RP are limited, but they have hinted at differences in the rate of organ-confined disease and surgical margin status, but not in grade reclassification rate [13]. Age, PSAD, and the number of positive cores are independent predictors of APFs in mixed models. Accordingly, PSAD predicts APF in patients undergoing immediate RP [14] and AS discontinuation [15,16], whereas elder patients frequently show a more aggressive disease [17]. Baseline age and PSA value were significantly different between LR and VLR groups. Unexpectedly, VLR men were older than LR ones. It could be hypothesized that diagnosis could follow screening in VLR cases whereas it is a consequence of clinical signs for LR ones. Moreover, those GAP3 patients undergoing radiation therapy were actually older than those undergoing surgery (Supplementary Table S3). This prevents us from strongly supporting such a hypothesis, although patients’ characteristics and tumor stage may influence both immediate and delayed radical treatment. Sayyid et al. recently reported that non-clinical parameters, as socio-demographic factors, are associated with the likelihood of undergoing active treatment among LR patients following AS [18].

LR patients underwent a more intensive biopsy schedule, including earlier first re-biopsy, and had more biopsy sets during surveillance than VLR patients. Although the number of biopsy sets is associated with risk of APFs, it remains unclear if the frequency of examinations could be a consequence of un-measured clinical features that induced clinicians to personalize the timing of biopsies. Different PSA kinetics may differently trigger extra-biopsies or treatment. Moreover, patients with no cancer at the first surveillance biopsy underwent less biopsy sets as well as had a lower rate of APFs. Tumor absence in the re-staging biopsy has been confirmed to predict AS outcome [15].

The main study strength is represented by large and composite numbers from world-wide expert centers that recruit hundreds of patients, permitting to have a real-world scenario of AS in prostatic cancer. Consequently, limitations include those inherent of such a nature and design. First, it is unclear to which extent these findings express the well-known imprecision of random prostate biopsy since the diagnosis. A sampling error occurs in 13.1% to 50% of men having cancer suitable for AS undergoing immediate RP [14,19,20,21,22], and our APF rate of 39% is within this range. The use of multi-parametric MRI and fusion-target biopsy might mitigate misclassification by identifying clinically significant cancers with a sensibility up to 95% [23] and decrease APF rate, when compared with standard biopsy [24,25]. PRIAS protocol introduced multi-parametric MRI since 2016 [8]. Unfortunately, few patients (6%) in the present dataset underwent MRI, preventing any inference, due to exiguity.

Second, the high proportion of missing data about the reason for leaving AS confines the generalization of our findings. Moreover, our findings focused on selected patients undergoing radical prostatectomy, and we cannot exclude possible biases due to treatment choice. Indeed, patients undergoing radiation therapy (Supplementary Table S3) were older and had clinical features closer to low-risk than to very-low-risk operated patients.

Third, we included positive surgical margins as an adverse outcome, even though we recognize the limitations of this parameter. Indeed, positive margins may be more dependent on the surgical technique rather than representing a biomarker of aggressiveness. The recorded rate eventually reveals the real life picture of such a heterogeneous cohort. Actually, positive margins have been seen as an unfavorable event in some instances, and not in others [13,22,26].

Fourth, we could not benefit from molecular biomarkers that may improve risk allocation over current clinical models by acting independently of tumor grading and risk group [27].

Finally, we lacked information on follow-up after surgery to assess any difference in harder outcomes according to APFs. Data on biochemical recurrence status will possibly extend the range of our findings. APFs stand as possible drivers for postoperative strategies, but their prognostic meaning in patients undergoing RP after AS remains unclear [28].

A review by Van den Bergh et al. found that curative treatment delay from months to even years were not associated with worse outcomes in men with LR prostatic cancer [29]. A more recent review found weak evidence of a higher risk of biochemical recurrence and worse pathological outcomes when surgery is delayed between 6 and 9 months, whereas worse survival outcome findings were not conclusive as an effect of delays beyond 12 months both for patients with intermediate risk and high risk PCa [30].

Finally, a recent series compared adverse pathology and the recurrence-free survival of patients undergoing radical prostatectomy after biopsy progression during AS. They did not find any difference between early (≤6 months) and late (6 months-5 years) neither in adverse pathology (all features) rate nor in 3-years recurrence-free survival [31].

## 5. Conclusions

Different AS entry criteria are significantly associated with the rate and timing of non-organ confined disease and with margin status but not with upgrading to GG3 or higher. Some parameters, namely PSAD and age, show a strong association with APFs and may underline a possible selection process.

AS stands as a safe option in men with LR prostate cancer, but most of them still have favorable findings at definitive pathology. The implementation of biological and imaging drivers may improve selection both at entry and at exit time to restore the imbalance generated by overdiagnosis and to limit overtreatment. Further insights are needed to assess the prognostic impact of each APF of those patients undergoing delayed surgery.

## Figures and Tables

**Table 1:** Baseline characteristics of patients in very low risk and low risk sub-groups undergoing radical prostatectomy.

Characteristics	Very Low Risk Group	Low Risk Group	*p* Value
	(*n* = 721)	(*n* = 1021)	
Age, *n* (%)			0.042
≤55 years	83 (11.5)	158 (15.5)	
56–60 years	149 (20.7)	233 (22.8)	
61–65 years	237 (32.9)	297 (29.1)	
66–70 years	184 (25.5)	236 (23.1)	
71–80 years	67 (9.3)	97 (9.5)	
>80 years	0 (0.0)	0 (0.0)	
Missing	1 (0.1)	0 (0.0)	
Median age (IQR), years	63.0 (8.0)	63.0 (9.0)	
PSA level, *n* (%)			<0.001
≤3.0 ng/ml	62 (8.6	65 (6.4)	
3.1–6.0 ng/ml	463 (64.2)	508 (49.8)	
6.1–10.0 ng/ml	196 (27.2)	448 (43.9)	
Median PSA level (IQR), ng/ml	4.90 (2.30)	5.70 (2.50)	
PSA density, *n* (%)			groups criterion
<0.15 ng/mL/cm^3^	721 (100)	242 (35.0)	
≥0.15 ng/mL/cm^3^	0 (0.0)	779 (65.0)	
Median PSA density (IQR), ng/mL/cm^3^	0.10 (0.04)	0.17 (0.05)	
Clinical T stage, *n* (%)			groups criterion
1	721 (100)	758 (74.2)	
2	0 (0.0)	263 (25.8)	
Number of positive cores, *n* (%)			<0.001
0	4 (0.6)	1 (0.1)	
1	493 (68.4)	458 (44.9)	
2	224 (31.1)	255 (25.0)	
≥3	0 (0.0)	129 (12.6)	
Missing	0 (0.0)	48 (4.7)	
Median Number of positive cores ± IQR	1 (1)	2 (2)	
Charlson Comorbidity Index, *n* (%)			0.086
0	117 (16.2)	176 (17.2)	
1	37 (5.1)	45 (4.4)	
2	57 (7.9)	52 (5.1)	
≥3	24 (3.3)	21 (2.1)	
Missing	486 (67.4)	727 (71.2)	
Ethnicity, *n* (%)			0.306
Hispanic or Latino	1 (0.14)	10 (1.0)	
Non-Hispanic	162 (22.47)	410 (40.2)	
Missing	558 (77.39)	601 (58.9)	
Smoking history, *n* (%)			0.208
Never	90 (12.48)	167 (16.4)	
Former	44 (6.19)	64 (6.3)	
Current	23 (3.19)	58 (5.7)	
Missing	564 (78.22)	732 (71.7)	
Reason for discontinuing AS, *n* (%)			0.188
Protocol reasons	420 (58.3)	632 (61.9)	
Without evidence of progression	60 (8.3)	66 (6.5)	
Other/unknown	241 (33.4)	323 (31.6)	

Abbreviations: SD: standard deviation; IQR: interquartile range; PSA Prostate Specific Antigen; AS Active Surveillance.

**Table 2:** Comparison of adverse pathological features between very low risk and low risk sub-group.

Characteristics	Very Low Risk	Low Risk	Odds Ratio	*p* Value
	(*n* = 721)	(*n* = 1021)		
Overall APFs	228 (31.7%)	455 (44.6%)	1.54 (1.24, 1.91)	<0.001
Pathological T stage ≥ pT3	111/568 (19.5%)	246/861 (28.6%)	1.47 (1.12, 1.93)	0.013
GG ≥ 3	124/569 (21.8%)	184/862 (21.4%)	0.95 (0.72, 1.24)	0.692
Positive surgical margins	81/462 (17.5%)	210/769 (27.3%)	1.80 (1.33, 2.45)	<0.001
Positive nodes	11/486 (2.3%)	15/753 (2.0%)	0.79 (0.34, 1.88)	0.582

*p* values for mixed effect models are reported. APF: adverse pathological findings; CIs: 95% confidence intervals.

**Table 3:** Univariable (A) and multivariable (B,C) analyses for adverse pathological findings.

**A: Univariable Analysis of Mixed Effects Model**		
**Variables of Model**	**Odds Ratio**	***p*** **Value**
Sub-group (Compared to Very low sub-group)		
Low	1.46 (1.15, 1.86)	<0.001
Age, years	1.04 (1.02, 1.06)	<0.001
PSA level, ng/mL	1.07 (1.01, 1.14)	0.029
PSA density, ng/mL/cm^3^	16.62 (2.96, 98.67)	<0.001
Number of biopsy cores with prostate cancer	1.16 (1.05, 1.29)	<0.001
Last biopsy time since AS, months	1.01 (1.00, 1.01)	0.015
Number of biopsies	1.22 (1.07, 1.40)	<0.001
Reasons leaving AS (Compare to Protocol reasons)		
No evidence of progression	0.44 (0.22, 0.84)	0.016
Others	0.54 (0.37, 0.76)	0 < 0.001
**B: Multivariable analysis of mixed effects model**		
**Variables of Model**	**Odds Ratio**	***p*** **Value**
Sub-group (Compared to Very low sub-group)		
Low	1.37 (1.07, 1.76)	0.014
Age, years	1.05 (1.06, 1.07)	<0.001
PSA level, ng/mL	1.04 (0.98, 1.11)	0.180
Last biopsy time since AS, months	1.01 (1.00, 1.01)	0.152
Number of biopsies	1.14 (0.95, 1.37)	0.166
Reasons leaving AS (Compare to Protocol reasons)		
No evidence of progression	0.48 (0.24, 0.92)	0.034
Others	0.61 (0.42, 0.88)	0.009
**C: Multivariable analysis of mixed effects model including PSA density and number of biopsy cores containing cancer (sub-group excluded)**		
**Variables of Model**	**Odds Ratio**	***p*** **Value**
Age, years	1.05 (1.03, 1.07)	<0.001
PSA level, ng/mL	0.99 (0.92, 1.07)	0.862
Last biopsy time since AS, months	1.00 (1.00, 1.01)	0.361
Number of biopsies	1.20 (0.99, 1.47)	0.069
PSA density, ng/mL/cm^3^	23.21 (3.01, 196.12)	0.003
Number of biopsy cores with prostate cancer	1.16 (1.05, 1.29)	0.004
Reasons leaving AS (Compare to Protocol reasons)		
No evidence of progression	0.53 (0.26, 1.00)	0.061
Others	0.67 (0.46, 0.96)	0.031

CIs: 95% confidence intervals.

## Data Availability

Within the GAP3 consortium, we agreed that the data cannot be shared without the approval of all parties.

## Supplementary Materials

The following supporting information can be downloaded at: https://www.mdpi.com/article/10.3390/cancers14153558/s1, Table S1: Baseline characteristics of patients undergoing radical prostatectomy after Active Surveillance (N 3000). Table S2: Characteristics of pathological findings for patients who had radical prostatectomy (N 3000). Table S3: Characteristics of patients from the whole GAP3 cohort who underwent external beam radiotherapy with or without androgen deprivation therapy (ADT) as radical treatment, after AS drop-out (N 2103). Table S4: Description of number and timing of biopsies during AS. Figure S1: Favorable (blue) and adverse (red) pathological findings frequency according to number of biopsy during AS in VLR and LR sub-groups. Figure S2: Distribution of patients (%) included in active surveillance per year of diagnosis, from the whole GAP3 cohort. Figure S3: Distribution of baseline PSA density values of very low-risk and low-risk patients, undergoing radical prostatectomy after active surveillance.

## Appendix A

Members of The Movember Foundation’s Global Action Plan Prostate Cancer Active Surveillance (GAP3) consortium

Principle Investigators: Bruce Trock (Johns Hopkins University, The James Buchanan Brady Urological Institute, Baltimore, MD, USA), Behfar Ehdaie (Memorial Sloan Kettering Cancer Center, New York, NY, USA), Peter Carroll (University of California San Francisco, San Francisco, CA, USA), Christopher Filson (Emory University School of Medicine, Winship Cancer Institute, Atlanta, GA, USA), Christopher Logothetis (MD Anderson Cancer Centre, Houston, TX, USA), Todd Morgan (University of Michigan and Michigan Urological Surgery Improvement Collaborative (MUSIC), Michigan, MI, USA), Laurence Klotz (University of Toronto, Sunnybrook Health Sciences Centre, Toronto, ON, Canada), Tom Pickles (University of British Columbia, BC Cancer Agency, Vancouver, BC, Canada), Eric Hyndman (University of Calgary, Southern Alberta Institute of Urology, Calgary, AB, Canada), Caroline Moore (University College London & University College London Hospital Trust, London, UK), Vincent Gnanapragasam (University of Cambridge & Cambridge University Hospitals NHS Foundation Trust, Cambridge, UK), Mieke Van Hemelrijck (King’s College London, London, UK & Guy’s and St Thomas’ NHS Foundation Trust, London, UK), Prokar Dasgupta (Guy’s and St Thomas’ NHS Foundation Trust, London, UK), Chris Bangma (Erasmus Medical Center, Rotterdam, The Netherlands/representative of Prostate cancer Research International Active Surveillance (PRIAS) consortium), Monique Roobol (Erasmus Medical Center, Rotterdam, The Netherlands/representative of Prostate cancer Research International Active Surveillance (PRIAS) consortium), The PRIAS study group, Arnauld Villers (Lille University Hospital Center, Lille, France), Grégoire Robert (Centre Hospitalier Universitaire de Bordeaux (CHU), Bordeaux, France), Axel Semjonow (University Hospital Muenster, Muenster, Germany), Antti Rannikko (Helsinki University and Helsinki University Hospital, Helsinki, Finland), Riccardo Valdagni (Department of Oncology and Hemato-oncology, Università degli Studi di Milano, Radiation Oncology 1 and Prostate Cancer Program, Fondazione IRCCS Istituto Nazionale dei Tumori, Milan, Italy), Antoinette Perry (University College Dublin, Dublin, Ireland), Jonas Hugosson (Sahlgrenska University Hospital, Göteborg, Sweden), Jose Rubio-Briones (Instituto Valenciano de Oncología, Valencia, Spain), Anders Bjartell (Skåne University Hospital, Malmö, Sweden), Lukas Hefermehl (Kantonsspital Baden, Baden, Switzerland), Lee Lui Shiong (Singapore General Hospital, Singapore, Singapore), Mark Frydenberg (Monash University; Cabrini Institute, Cabrini Health, Melbourne, Australia), Phillip Stricker (St Vincents Prostate Cancer Centre, Sydney, Australia), Mikio Sugimoto (Kagawa University Faculty of Medicine, Kagawa, Japan), Byung Ha Chung (Gangnam Severance Hospital, Yonsei University Health System, Seoul, Korea).

Pathologist: Theo van der Kwast (Princess Margaret Cancer Centre, Toronto, ON, Canada).

Technology Research Partners: Wim van der Linden, Tim Hulsen, Boris Ruwe, Peter van Hooft (Royal Philips, Eindhoven, The Netherlands).

Executive Regional statisticians: Ewout Steyerberg (Erasmus Medical Center, Rotterdam, The Netherlands), Daan Nieboer (Erasmus Medical Center, Rotterdam, The Netherlands); Kerri Beckmann (King’s College London, London, UK & Guy’s and St Thomas’ NHS Foundation Trust, London, UK), Brian Denton (University of Michigan, Ann Arbor, Michigan, USA), Andrew Hayen (University of Technology Sydney, Australia), Paul Boutros (Ontario Institute of Cancer Research, Toronto, ON, Canada).

Clinical Research Partners’ IT Experts: Wei Guo (Johns Hopkins University, The James Buchanan Brady Urological Institute, Baltimore, MD, USA), Nicole Benfante (Memorial Sloan Kettering Cancer Center, New York, NY, USA), Janet Cowan (University of California San Francisco, San Francisco, CA, USA), Dattatraya Patil (Emory University School of Medicine, Winship Cancer Institute, Atlanta, GA, USA), Lauren Park (MD Anderson Cancer Centre, Houston, TX, USA), Stephanie Ferrante (University of Michigan and Michigan Urological Surgery Improvement Collaborative, Ann Arbor, MI, USA), Alexandre Mamedov (University of Toronto, Sunnybrook Health Sciences Centre, Toronto, ON, Canada), Vincent LaPointe (University of British Columbia, BC Cancer Agency, Vancouver, BC, Canada), Trafford Crump (University of Calgary, Southern Alberta Institute of Urology, Calgary, AB, Canada), Vasilis Stavrinides (University College London & University College London Hospital Trust, London, UK), Jenna Kimberly-Duffell (University of Cambridge & Cambridge University Hospitals NHS Foundation Trust, Cambridge, UK), Aida Santaolalla (King’s College London, London, UK & Guy’s and St Thomas’ NHS Foundation Trust, London, UK), Daan Nieboer (Erasmus Medical Center, Rotterdam, The Netherlands), Jonathan Olivier (Lille University Hospital Center, Lille, & Centre Hospitalier Universitaire de Bordeaux (CHU), Bordeaux France), Tiziana Rancati (Fondazione IRCCS Istituto Nazionale dei Tumori di Milano, Milan, Italy), Helén Ahlgren (Sahlgrenska University Hospital, Göteborg, Sweden), Juanma Mascarós (Instituto Valenciano de Oncología, Valencia, Spain), Annica Löfgren (Skåne University Hospital, Malmö, Sweden), Kurt Lehmann (Kantonsspital Baden, Baden, Switzerland), Catherine Han Lin (Monash University and Epworth HealthCare, Melbourne, Australia), Thomas Cusick (St Vincents Prostate Cancer Centre, Sydney, Australia), Hiromi Hirama (Kagawa University, Kagawa, Japan), Kwang Suk Lee (Yonsei University College of Medicine, Gangnam Severance Hospital, Seoul, Korea).

Research Advisory Committee: Guido Jenster (Erasmus MC, Rotterdam, The Netherlands), Anssi Auvinen (University of Tampere, Tampere, Finland), Anders Bjartell (Skåne University Hospital, Malmö, Sweden), Masoom Haider (University of Toronto, Toronto, ON, Canada), Kees van Bochove (The Hyve B.V. Utrecht, Utrecht, The Netherlands).

Management team: Michelle Kouspou (Movember Foundation, Melbourne, Australia), Kellie Paich (Movember Foundation, Los Angeles, CA, USA), Chris Bangma (Erasmus Medical Center, Rotterdam, The Netherlands), Monique Roobol (Erasmus Medical Center, Rotterdam, The Netherlands), Jozien Helleman (Erasmus Medical Center, Rotterdam, The Netherlands).
