# Supplementary material for: Adverse Pathological Findings at Radical Prostatectomy following Active Surveillance: Results from the Movember GAP3 Cohort
Source: Cancers (Basel). 2022 Jul 22;14(15):3558. doi: 10.3390/cancers14153558 (PMC9332009; doi:10.3390/cancers14153558)

Supplementary

The supplementary material describes the characteristics of all the patients from the GAP3 cohort undergoing radical treatment (radical prostatectomy or radiation therapy) after dropping out of active surveillance (AS).

Supplementary Tables S1-3 describe the characteristics of all the patients from the whole GAP3 cohort undergoing radical treatment (radical prostatectomy or radiation therapy) after dropping out of active surveillance (AS).

Supplementary Table S4 and Figure S1 describe the timing and number of re-biopsies according to risk sub-group in the target population of very low and low risk patients undergoing radical prostatectomy.

Supplementary Figure S2 displays the proportion of patients enrolled in the GAP3 active surveillance cohort per year of diagnosis.

Supplementary Figure S3 displays PSA density distribution in very low and low risk patients undergoing radical prostatectomy.

Table S1. Baseline characteristics of patients undergoing radical prostatectomy after Active Surveillance (N 3000)

	N (%)<br>3000
**Grade Group (Gleason Score)**	
**1 (3+3)**	2680 (89.3)
**2 (3+4)**	229 (7.6)
**3 (4+3)**	31 (1.0)
**4 (sum score 8)**	9 (0.3)
**5 (sum score 9-10)**	1 (0.1)
**Missing**	50 (1.7)
**PSA at diagnosis, ng/mL**	
**≤3.0**	247 (8.2)
**3.1-6.0**	1496 (49.9)
**6.1-10.0**	932 (31.2)
**>10.0**	189 (6.3)
**Missing**	136 (4.5)
**Clinical T stage (cT)**	
**T1**	2174 (72.5)
**T2**	442 (14.7)
**T3**	1 (0.1)
**T4**	0 (0.0)
**Unknown**	10 (0.3)
**Missing**	373 (12.5)
**Number of positive cores at biopsy**	
**0**	9 (0.3)
**1**	1504 (50.2)
**2**	759 (25.3)
**≥3**	601 (20.0)
**Missing**	127 (4.2)
**Median positive cores (IQR)**	1 (1-2)
**PSA density ng/mL/cm^3**	
**<0.15**	1269 (42.3)
**≥0.15**	1035 (34.5)
**Missing**	696 (23.2)
**Median PSA density (IQR)**	0.14 (0.10-0.18)

Abbreviations: *PSA* Prostatic Specific Antigen; *IQR* interquartile range.

Table S2. Characteristics of pathological findings for patients who had radical prostatectomy (N 3000)

	N (%)<br>3000
**Grade Group (Gleason Score)**	
**1 (3+3)**	625 (20.8)
**2 (3+4)**	1182 (39.4)
**3 (4+3)**	363 (12.1)
**4 (sum score 8)**	65 (2.2)
**5 (sum score 9-10)**	91 (3.0)
**Missing**	674 (22.5)
**Pathological T-stage (pT)**	
**T0**	5 (0.2)
**T1**	0 (0.0)
**T2**	1720 (57.5)
**T3**	615 (20.5)
**T4**	3 (0.1)
**Unknown**	5 (0.2)
**Missing**	652 (21.7)
**Surgical margins**	
**Negative**	1492 (49.7)
**Positive**	296 (9.9)
**Unifocal**	75 (2.5)
**Multifocal**	70 (2.3)
**Unknown**	5 (0.2)
**Missing**	1062 (35.4)
**Pathological lymph node status (pN)**	
**N0**	1346 (44.9)
**N1**	46 (1.5)
**NX**	623 (20.8)
**Missing**	985 (32.8)

Table S3. Characteristics of patients from the whole GAP3 cohort who underwent external beam radiotherapy with or without androgen deprivation therapy (ADT) as radical treatment, after AS drop-out (N 2103)

	**N (%) (2103)**
**Age (years)**	
**≤ 55**	103 (4.9)
**56-60**	206 (9.8)
**61-65**	464 (22.1)
**66-70**	659 (31.3)
**71-80**	655 (31.1)
**> 80**	14 (0.7)
**Missing**	2 (0.1)
**Mean age ± SD,**	67.12 ± 6.46
**Median age (IQR)**	68.0 (9.0)
**PSA level, ng/ml**	
**≤ 3.0**	147 (7.0)
**3.1-6.0**	1011 (48.1)
**6.1-10.0**	716 (34.0)
**>10.0**	138 (6.6)
**Missing**	91 (4.3)
**Mean PSA level ± SD**	6.23 ± 3.33
**Median PSA level (IQR)**	5.70 (2.88)
**PSA density, ng/ml/cm^3**	
**< 0.15**	873 (41.5)
**≥ 0.15**	754 (35.9)
**Missing**	476 (22.6)
**Mean PSA density ± SD**	0.16 ± 0.11
**Median PSA density (IQR)**	0.14 (0.08)
**Number of positive cores**	
**0**	5 (0.2)
**1**	1039 (49.4)
**2**	582 (27.7)
**≥ 3**	391 (18.6)
**Missing**	86 (4.1)
**Median number of positive cores (IQR)**	1 (1)
**Reason for discontinuing AS**	
**Protocol reasons**	1338 (63.6)
**Without evidence of progression**	190 (9.1)
**Other/Unknown**	575 (27.3)

Abbreviations: *PSA* Prostatic Specific Antigen; *AS* Active Surveillance; *SD* standard deviation; *IQR* interquartile range.

Table S4: Description of number and timing of biopsies during AS

	Very low risk sub-group (N = 527)	Low risk sub-group (N = 797)	Total (N = 1324)	P values
**Time from diagnosis to first AS biopsy, n (%)**				< 0.001
**<1 month**	84 (15.9)	271 (34.0)	355 (26.8)	
**1-12 months**	270 (51.2)	303 (38.0)	573 (43.3)	
**13-24 months**	146 (27.7)	189 (23.7)	335 (25.3)	
**>24 months**	27 (5.1)	34 (4.3)	61 (4.6)	
**Median time to first AS biopsy (IQR), months**	12 (6-13)	11 (0-13)	12 (0-13)	
**Cancer present in first AS biopsy, n (%)**				< 0.001
**No**	240 (45.5)	259 (32.5)	499 (40.0)	
**Yes**	169 (32.1)	361 (45.3)	530 (37.7)	
**Missing**	118 (22.4)	177 (22.2)	295 (22.3)	
**Total number of biopsies during AS, n (%)**				0.051
**1**	322 (60.9)	429 (53.8)	751 (56.7)	
**2**	120 (22.6)	228 (28.6)	348 (26.3)	
**3**	60 (11.3)	88 (11.0)	148 (11.2)	
**4**	15 (2.5)	26 (3.3)	41 (3.1)	
**≥ 5**	10 (1.9)	26 (3.3)	36 (2.7)	
**Median number of biopsies (IQR), months**	1 (1-2)	1 (1-2)	1 (1-2)	

Abbreviations: IQR: interquartile range

Figure S1: Favorable (blue) and adverse (red) pathological findings frequency according to number of biopsies during AS in VLR and LR sub-groups.

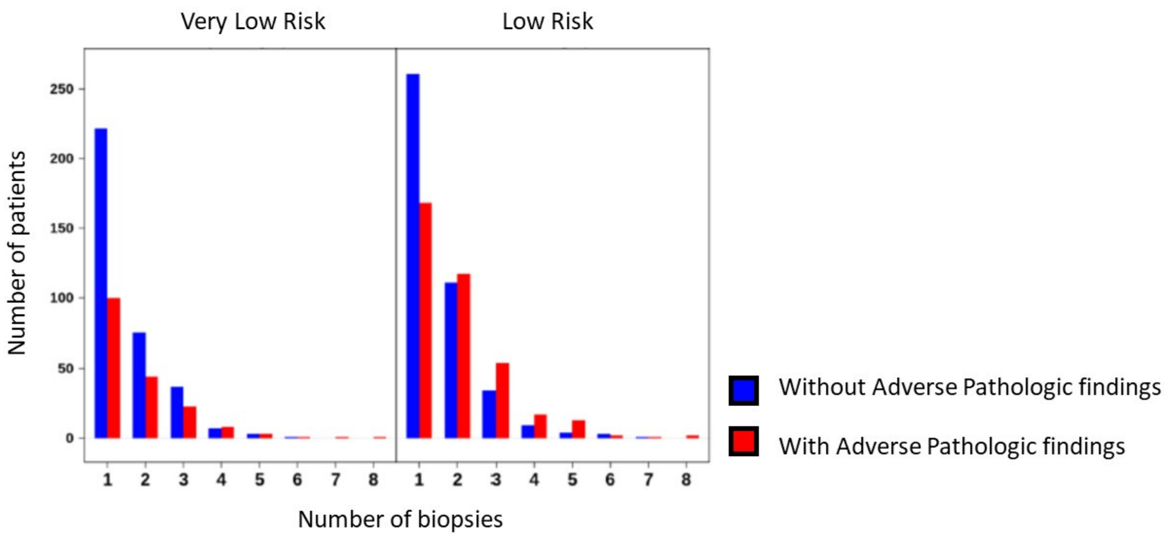

Figure S2: Distribution of patients (%) included in active surveillance per year of diagnosis, from the whole GAP3 cohort.

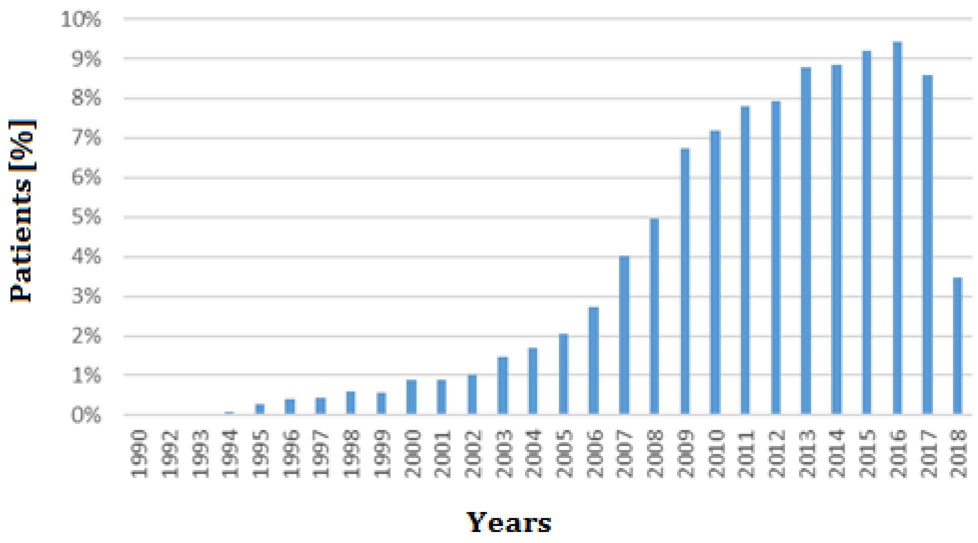

Figure S3: Distribution of baseline PSA density values of very low risk and low risk patients, undergoing radical prostatectomy after active surveillance.

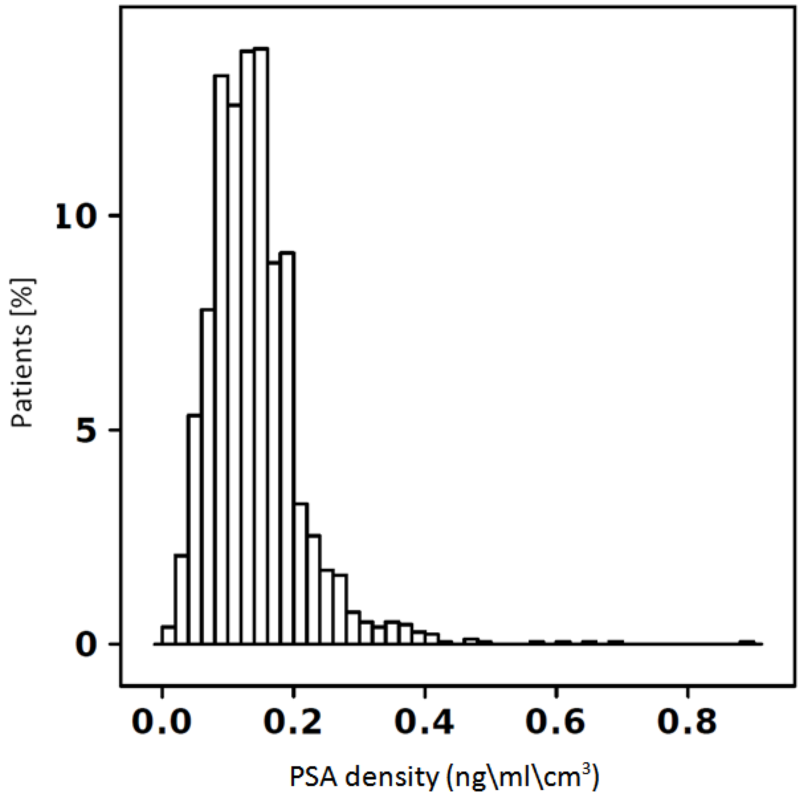

Supplement: Supplementary file 1 [file cancers-14-03558-s001.zip › cancers-1792669-supplementary.pdf]
